# Quantitation of Murine Stroma and Selective Purification of the Human Tumor Component of Patient-Derived Xenografts for Genomic Analysis

**DOI:** 10.1371/journal.pone.0160587

**Published:** 2016-09-09

**Authors:** Valentina E. Schneeberger, Viola Allaj, Eric E. Gardner, J. T. Poirier, Charles M. Rudin

**Affiliations:** 1 Molecular Pharmacology & Chemistry Program and Department of Medicine, Memorial Sloan Kettering Cancer Center, New York, NY, United States of America; 2 Pharmacology Graduate Training Program, Johns Hopkins University, Baltimore, MD, United States of America; 3 Department of Medicine, Weill Cornell Medical College, New York, NY, United States of America; New York University School of Medicine, UNITED STATES

## Abstract

Patient-derived xenograft (PDX) mouse models are increasingly used for preclinical therapeutic testing of human cancer. A limitation in molecular and genetic characterization of PDX tumors is the presence of integral murine stroma. This is particularly problematic for genomic sequencing of PDX models. Rapid and dependable approaches for quantitating stromal content and purifying the malignant human component of these tumors are needed. We used a recently developed technique exploiting species-specific polymerase chain reaction (PCR) amplicon length (ssPAL) differences to define the fractional composition of murine and human DNA, which was proportional to the fractional composition of cells in a series of lung cancer PDX lines. We compared four methods of human cancer cell isolation: fluorescence-activated cell sorting (FACS), an immunomagnetic mouse cell depletion (MCD) approach, and two distinct EpCAM-based immunomagnetic positive selection methods. We further analyzed DNA extracted from the resulting enriched human cancer cells by targeted sequencing using a clinically validated multi-gene panel. Stromal content varied widely among tumors of similar histology, but appeared stable over multiple serial tumor passages of an individual model. FACS and MCD were superior to either positive selection approach, especially in cases of high stromal content, and consistently allowed high quality human-specific genomic profiling. ssPAL is a dependable approach to quantitation of murine stromal content, and MCD is a simple, efficient, and high yield approach to human cancer cell isolation for genomic analysis of PDX tumors.

## Introduction

Since the early 1900’s, mice have emerged as the species of choice for cancer research due to their high breeding potential, low cost, small size, and ease of genetic manipulation. Multiple techniques have been developed to design relevant mouse models that fit the needs of research. Common approaches include genetically engineered mouse models (GEMMs) which recapitulate malignancy through targeted modifications of driver oncogenes and tumor suppressors, carcinogen induced tumor models, and heterotopic or orthotopic injection of human cancer cell lines in immunocompromised strains [1, 2]. While each of these mouse models offer unique advantages, they also display significant limitations that hinder their reliability as experimental models.

Oncogenesis in GEMMs is driven by selective targeted alteration of a small number of genes, typically one to three, yielding tumors that are not of human origin, and which may poorly reflect the genetic complexity and heterogeneity of human tumors. Carcinogen-induced mouse models may have increased heterogeneity and genetic complexity, but are also not of human origin, may not reflect relevant carcinogenic exposures, and their sporadic derivation and long latency markedly limits their utility as a preclinical platform for therapeutic research. Traditional cell line xenograft models are based on implantation of human cancer cell lines established and maintained *in vitro* into immunosuppressed mice. Establishment of a cancer cell line *in vitro* has been shown to reduce heterogeneity of the cell population [3, 4], in part due to selection of subclones with consistent proliferative capacity under tissue culture conditions that differ greatly from the *in vivo* environment: typically two-dimensional growth on plastic, in high oxygen tension and high glucose media, where tissue invasive capacity and angiogenic drive are not required. We and others have shown that the selective pressure for cell line establishment leads to substantial epigenetic and gene expression changes relative to the tumors from which they were derived, changes that are not reversed by implantation into mice [5, 6]. While extensively used, cell line xenograft models can be unreliable predictors of drug efficacy, with compounds that performed well in mouse models failing when translated to human clinical trials [7, 8].

Patient derived xenografts are based on rapid transfer of human tumor tissue from a patient into immunosuppressed mice, with no intervening *ex vivo* cell culture [9]. Relative to cell line-based xenografts, PDX better retain the gene expression profiles, epigenetic landscape, tumor heterogeneity, and biological characteristics of the human tumors of origin [10, 11]. PDX models also have major limitations: most notably, like human cell line xenografts, their establishment generally requires that the murine host be profoundly immunodeficient, largely precluding use of these models to evaluate novel immunotherapies. Mice entirely lacking an adaptive immune system are at risk for developing spontaneous malignancies, which in some cases may resemble xenograft tumors leading to extensive waste of time and resources spent studying the wrong cancer type [12]. Despite these and other caveats, PDX models have become increasingly popular as a platform for preclinical therapeutic testing, based on a growing body of evidence that they may better reflect the clinical activity of anti-cancer therapeutics than cell line xenografts [13, 14].

Although the malignant human clone is retained during establishment and passaging of a PDX tumor model, the tumor-associated stroma is replaced by corresponding murine stromal components: extracellular matrix, cancer-associated fibroblasts, endothelial and perivascular cells, macrophages, and others [15]. The complex direct and paracrine interactions between cancer cells and stromal elements that comprise a tumor are areas of active investigation, and have been shown to be essential regulators of tumor growth [16–18]. The relative composition of cancer cells and supporting stromal elements is highly variable among different cancer types, and among cancers of a single histologic type and tissue of origin. In many tumor types, notably pancreatic adenocarcinoma, the malignant clone may represent a small minority of the cells within tumors [19]. Accurate estimation of the cellular fraction of a tumor composed of the malignant clone vs. non-malignant stromal elements is difficult in human tumors and in GEMM models, as all the cells are of the same species of origin. PDX models offer an opportunity to track the stromal fraction of tumors over time, and under different conditions. This may be of particular relevance for studying the effects of classes of agents that target tumor-associated stromal components, such as vascular disrupting agents and factors contributing to production of dense, extracellular tumor matrices [20–22].

The admixture of human and mouse cells in a PDX tumor interferes with genomic characterization of the human component of these tumors. The presence of mouse DNA in human-directed next generation sequencing can lead to identification of false positive single nucleotide variants from reads that map to both the human and mouse reference genomes and overall loss of sequencing depth, limiting power to detect low frequency or subclonal mutations. Bioinformatic approaches can partially remedy this problem, but are not sufficient for tumors with high stromal content and in particular may bias toward false negative calls by eliminating human sequences with true mutations in regions of high homology to the mouse genome [23–25]. On the other hand, the admixture of human cancer and murine stroma in PDX tumors offers an opportunity, relative to direct analysis of human tumors, for deep sequencing of the purified malignant component, after species-specific separation of mouse and human cells. High purity separation of human cancer cells from human stroma is substantially more challenging.

The Eshleman laboratory recently defined an approach to quantitate murine and human content of xenograft tumors based on PCR amplification of orthologous regions of the murine and human genome that differ slightly in length, a technique we are here referring to as species-specific PCR amplicon length (ssPAL) analysis [26]. Using fluorescently tagged PCR primers to amplify these regions followed by capillary electrophoresis, the percentage of murine tissue contamination can be accurately calculated based on fragment sizing. Advantages of this technique include its simplicity, accuracy, low cost, and limited requirements for starting material.

In this paper, we applied ssPAL as a benchmark to quantitatively assess the baseline human cancer cell content in a series of human lung cancer PDX lines generated by our group. We further evaluated the changes in tumor stromal content in several PDX lines over multiple generations to assess the stability of this tumor feature through multiple passages.

We then compared the performance of four methodologies to separate the human and murine cells from 5 PDX lines, using ssPAL analysis to assess the resulting human cancer cell purity. Germline DNA, total tumor DNA, and purified human cancer cell DNA generated from one PDX using each of the four separation techniques was subjected to next generation sequencing using MSK-IMPACT, a clinically validated exon capture targeted sequencing approach evaluating 341 cancer-associated genes [27]. All samples were sequenced to >800× average depth of coverage (DoC).

We report here that the combination of ssPAL analysis and mouse cell depletion by immunomagnetic bead separation (negative selection) defines a simple, dependable and useful approach to address multiple issues of relevance to PDX mouse models, including serial analysis of stromal content, identification and avoidance of spontaneously arising contaminating murine tumors, and high purity isolation of human tumor DNA for detailed genomic mutational analysis.

## Materials and Methods

### Samples and Genomic DNA extraction

Tumor samples were obtained from PDX lines generated as described previously [5]. Briefly, for each PDX, patient tissue was collected in the clinic and transported to the laboratory in PBS at 4°C. The sample was then minced into fine pieces using a fresh razor blade, then passed through a 60 μm filter. Following centrifugation, the sample was resuspended in 100 μL of 50:50 HBSS:matrigel and injected subcutaneously in the flank of a NOD/SCID mouse. Each successful PDX line was assigned a unique ID. The lung cancer PDX lines used in this study were MSK-LX27, MSK-LX29, SCRX-LU149, JHU-LX1, JHU-LX110, JHU-LX33b, JHU-LX44, JHU-LX55a, MSK-LX40, MSK-LX25, MSK-LX68, MSK-LX13, MSK-LX95, MSK-LX96, MSK-LX97, MSK-LX59, MSK-LX242, and MSK-LX36. Tumor sampling for establishment of PDX lines was performed under a research protocol approved by the Memorial Sloan Kettering Institutional Review Board, and *in vivo* experimentation was performed under research protocols approved by the Memorial Sloan Kettering Institutional Animal Care and Use Committee. To obtain tumor samples used in study, tumor-bearing animals were euthanized with CO_2_ and subcutaneous flank tumors were excised, grossly removing any murine skin from the tumor tissue. Tumors usually do not infiltrate into the viscera and remain encapsulated, which facilitates the dissection. Approximately 300 mg of tumor tissue was placed into a gentleMACS^™^ C tube, containing 5 mL of serum-free RPMI-1640 media, supplemented with one aliquot of human tumor dissociation kit enzymes (Miltenyi). Tissue was enzymatically dissociated for one hour on a Miltenyi gentleMACS^™^ Octo Dissociator, running the “h_TDK1” standard cycle. Dissociated single cell suspensions were then poured through a 70 μm mesh filter to trap fibrous materials, quenching the enzymatic digestion with 25 mL of FACS buffer (PBS + 1% FBS, + 1 mM EDTA). Cells were pelleted at 300 rcf for 5 minutes, supernatant aspirated and then the cell pellet was re-suspended in 5ml of ACK lysis buffer (Crystalgen) and rotated at room temperature for 3 minutes. The cell suspension was again quenched with 25 mL of FACS buffer, pelleted and re-suspended; this procedure was repeated for a total of 3 washes post-ACK lysis. Cells were counted using trypan blue exclusion. DNA extraction was performed using the Qiagen DNeasy blood and tissue kit according to manufacturer protocol. Genomic human DNA from Jurkat cells (Thermo Scientific) and genomic mouse DNA from NIH3T3 cells (New England Biolabs) were used as controls.

### ssPAL analysis

ssPAL was performed as described previously using primer pair 43 and 5 [26]. Briefly, this technique uses PCR primers to amplify conserved regions of slightly differing lengths between the mouse and human genomes. Following capillary electrophoresis (Genewiz) and peak analysis using the Peak scanner software (Life Technologies) the amount of murine and human DNA in a given sample can be accurately quantified. Two primer pairs were used in this study. Primer pair 5 amplifies a region of the Ribonuclease P/MRP 38kDa subunit gene on chromosome 10p13 and yields a 272 bp long (human) PCR product and a 278 bp long (mouse) PCR product. The forward primer sequence is 5’-TCATTGGCTTAAAATGTGT-3’, and the reverse primer sequence is 5’-FAM-TTTATTTTAAGGGGTTGTAATG-3’. Primer pair 43 amplifies a region of the downstream ring finger and CCCH-type zinc finger domains 2 (RC3H2) on chromosome 9q34 and yields a 211 bp long (human) PCR product and a 206 bp long (mouse) PCR product. The forward primer sequence is 5’-CTATTCCTATAGCACAAAGG-3’, and the reverse primer sequence is 5’-FAM-GATGGTGTACACCCATCATG-3’. PCR conditions were as follows: 98°C for 5 minutes, 35 cycles of 98°C for 30 seconds, 52°C for 30 seconds and 72°C for 30 seconds followed by a final elongation step of 72°C for 10 minutes. Phusion high fidelity master mix (New England Biolabs) was used to prepare the PCR reaction. Resulting PCR products for each primer pair were diluted 1:32 in nuclease free water then mixed together in a 1:1 ratio before being sent out for capillary electrophoresis.

### Cell separation kits and FACS

The mouse cell depletion kit (Miltenyi), EpCAM positive cell selection kit (Miltenyi), and EasySep EpCAM positive cell selection kit (STEMCELL technologies) were used according to protocols. For FACS, 10 million cells were blocked in 1 mL of FACS buffer by the addition of 50 μL of Human TruStain FcX^™^ (BioLegend) and 25 μL of Mouse BD Fc Block^™^ (BD Pharmingen) for 20 minutes at room temperature, then stained for 1 hour at 4°C with 5 μL of PE mouse anti-mouse H-2K^d^ (1:200 dilution) (BD Pharmingen; clone SF1-1.1) and 5 μL of APC mouse anti-human CD326 (1:200 dilution) (Miltenyi; clone HEA-125). Single color staining dilutions were previously determined and single color controls were included in all experiments. Cells were washed 3 times with 5 mL of FACS buffer and then re-suspended in 1 mL of FACS counterstain buffer containing 200 ng/mL DAPI (Lonza) and 25 μg/mL DNAse I and DNAse I buffer additives (New England Biolabs). Cells were sorted on BD FACSAria II instrument in the MSKCC Flow Cytometry Core Facility.

### *In silico* simulation of virtual normal, virtual tumor, and virtual PDX

High depth whole genome shotgun sequencing (WGSS) data for HapMap individual NA12882 (study ERP001775) was downloaded from the European Nucleotide Archive (ENA) comprising two independent technical replicates. To generate a virtual normal, one NA12882 technical replicate was aligned to either the hg19 human reference genome or a hybrid human/mouse genome using BWA 0.7.12, then downsampled using SAMtools 1.2 to 30× DoC [28, 29]. Hybrid genome alignments were performed by aligning to a reference genome composed of all human and mouse contigs concatenated into a single file. Mouse contigs were excluded from all subsequent analysis. A second technical replicate was aligned to the hybrid genome, and downsampled to 60× DoC. To generate a virtual tumor, a completely known mutation profile was then introduced by BAM replacement using BAMSurgeon and the IS3 mutation set from the ICGC-TCGA DREAM Somatic Mutation Calling Challenge at a range of variant allele frequencies [30]. WGSS data from the NSG mouse strain generated by the The Wellcome Trust Sanger Institute Mouse Genomes Project was downloaded from the ENA (study ERP004378) and aligned to the hybrid genome [31]. Human reads from the virtual tumor sample were then randomly replaced with NSG mouse reads at a range of contamination levels using SAMtools. Mutations were called using MuTect, then compared to the IS3 mutation set to determine the precision, recall, and *F*-score, (F1, harmonic mean of precision and recall) [32].

## Results

### Murine stromal content varies significantly between PDX lines

**Fig 1:**
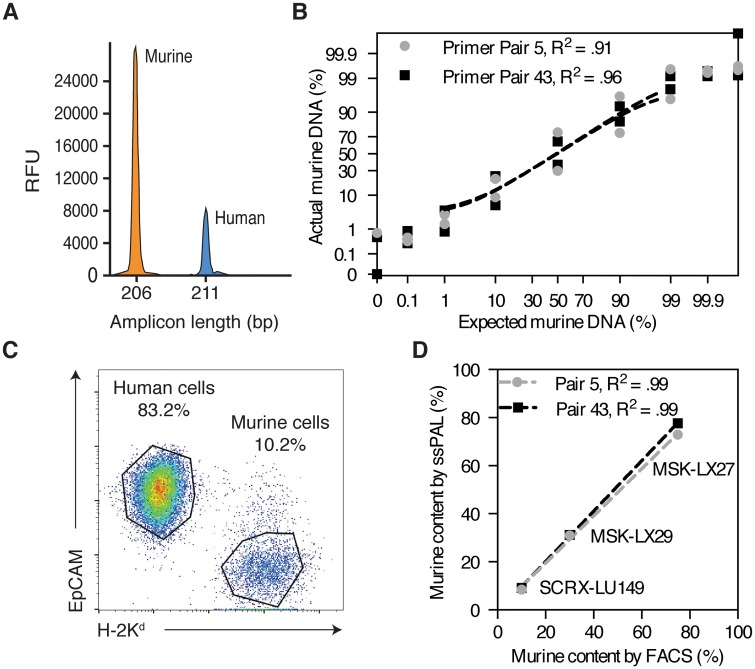
ssPAL analysis yields precise measurements with accuracy comparable to FACS. (A) After performing capillary electrophoresis, the presence of each PCR product (human and murine) for both primer pairs is evaluated. The peak at 206 bp corresponds to the murine fraction (orange), the peak at 211 bp correspond to the human fraction (blue). The resulting peak areas are proportional to the murine and human DNA content in a given sample. (B) ssPAL analysis is performed using two primer pairs (5 and 43) that amplify homologous regions of the mouse and human genome. This technique can accurately detect the percentages of murine DNA in pre-set mixtures of NIH 3T3 and Jurkat cells DNA within a range of 1% to 99%. Sensitivity is lost when analyzing values outside of this range. (C) FACS is the gold standard to separate human and murine cells and quantify the percentage of each population. In this representative plot, a PDX tumor from line MSK-LX29 is sorted using EpCAM and H-2K^d^. (D) Murine DNA content determined by ssPAL is proportional to murine cell content measured by FACS.

ssPAL analysis is performed using 2 pairs of PCR primers (Primer Pair 5 and Primer Pair 43), each of which target a distinct homologous locus of the mouse and human genome [26]. The PCR products are diluted 1:32 with nuclease free water then mixed at a 1:1 ratio. The resulting peak areas are proportional to the amount of mouse and human DNA template present in the PCR reaction Fig 1A. Each primer pair yields highly similar results Fig 1B. We prepared mixtures with different percentages of mouse and human genomic DNA to test the sensitivity limits of the ssPAL technique and confirmed the findings of Lin et al. that ssPAL analysis can detect as little as 1% mouse contamination and reliably identifies the known sample composition of each mixture across a broad range of DNA ratios Fig 1B. We then determined the accuracy of ssPAL analysis by comparing the calculated ssPAL percentages of murine stromal contamination for three of our PDX lines to percentages calculated using FACS. FACS permits separation and quantitation of human and murine fractions using species-specific antibodies (DAPI^-^, H-2K^d+^ for mouse stromal cells vs. DAPI^-^, EpCAM^+^ for human cancer cells) [33] Fig 1C. Both methods yielded nearly identical results, establishing ssPAL analysis as a tool comparable to FACS to quantify murine and human fractions in a tumor sample, but significantly less expensive, time consuming, and resource intensive Fig 1D.

**Fig 2:**
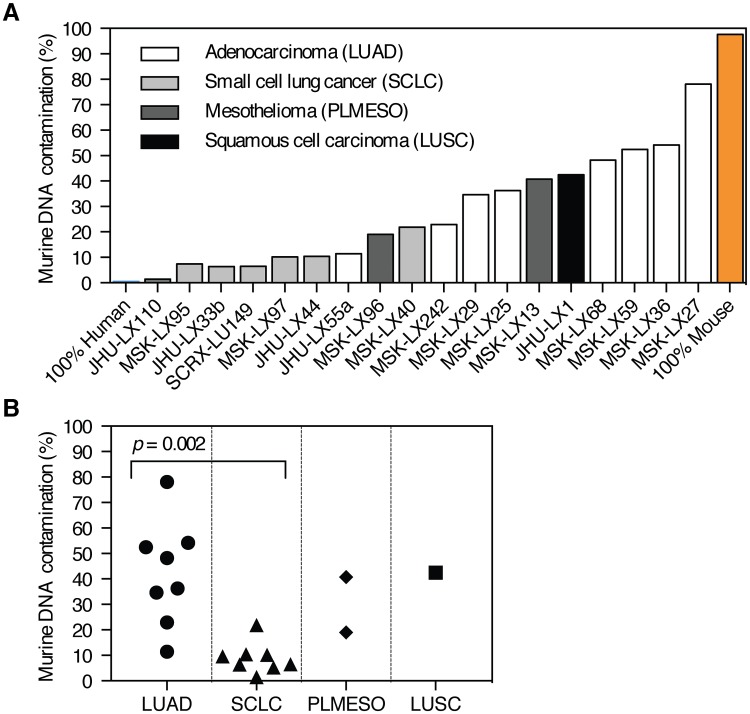
ssPAL analysis highlights significant murine stromal content variation across multiple lung PDX lines. (A) The ssPAL analysis results for primer pairs 5 and 43 were averaged. Murine stromal contamination exhibits a wide range between PDX lines. (B) While stromal contamination is variable between PDX lines, it is consistent between tumors of the same cancer subtype. Stromal content in SCLC PDX was significantly lower than in LUAD (two-tailed Student’s *t*-test). LUAD = lung adenocarcinoma, SCLC = small cell lung cancer, PLMESO = pleural mesothelioma, LUSC = lung squamous carcinoma.

We then extracted genomic DNA from flash frozen PDX tumor tissue to compare mouse stromal contamination across multiple PDX lines. ssPAL analysis detected significant stromal variation between lung PDX lines Fig 2A. Interestingly, we found that the amount of stromal contamination varied significantly between PDX of different histology and mirrored the observed trends in human primary tumors. Among the tumor types in this study, which included lung adenocarcinoma, small cell lung carcinoma, mesothelioma, and squamous cell carcinoma. Significantly less stromal contamination was present in small cell carcinoma as compared to lung adenocarcinoma, consistent with the WHO classification of this tumor as having little stromal content [34] Fig 2B.

**Fig 3:**
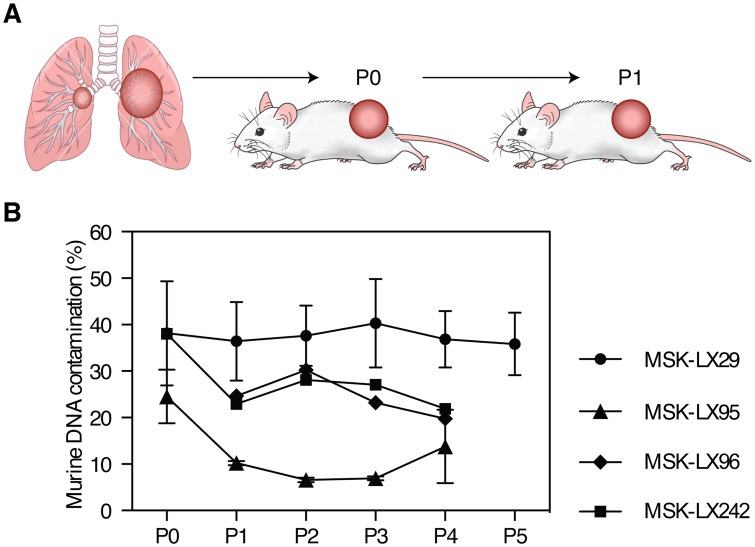
PDX mouse model tumor transplantation schema and passage over time. (A) This schematic summarizes our protocol for PDX generation, implantation, and passaging. Tumor tissue is collected from the patient and prepped into a single cell suspension using the gentleMACS^™^ Octo Dissociator. Cells are mixed 1:1 with matrigel and implanted single flank in immunosuppressed mice. Once tumor reaches end volume and is ready for passage; it is collected, processed into a single cell suspension, then cells are re-implanted in the next set of mice. (B) ssPAL analysis reveals that PDX tumors do not show significant variation in murine stromal content over successive passages. MSK-LX242 and MSK-LX29 are lung adenocarcinoma PDX lines, MSK-LX95 is a SCLC PDX line, and MSK-LX96 is a mesothelioma PDX line. Error bars represent standard error. P0 = Passage 0, P1 = Passage 1, P2 = Passage 2, etc.

During the process of primary tumor engraftment and expansion of resulting PDX lines for experimentation, tumors undergo several passages exclusively in mice Fig 3A. ssPAL analysis can track murine stromal fluctuations over successive passages. It is possible that the stromal content in a given tumor model is stochastic, and might vary substantially from passage to passage. Alternatively, the stroma:cancer cell ratio may represent a characteristic feature of the tumor model, and remain relatively constant. To evaluate these alternative possibilities, we performed ssPAL analysis for the PDX lines MSK-LX29 (LUAD), MSK-LX242 (LUAD), MSK-LX95 (SCLC), and MSK-LX96 (PLMESO) over successive passages post primary tumor engraftment. A single tumor was collected from each passage for DNA extraction and ssPAL analysis. The ssPAL analysis was repeated two times with the same starting materials and the results of primer pair 43 and 5 were averaged to yield a single curve. The percentage of mouse stroma remained within a remarkably narrow range over multiple passages Fig 3B. Interestingly, the murine composition for passage 0 of PDX line MSK-LX242 and MSK-LX95 is higher than the remaining passages; however, once the cells have adapted and are subsequently passaged, stromal content remains constant over multiple passages.

### Evaluation of separation techniques

**Fig 4:**
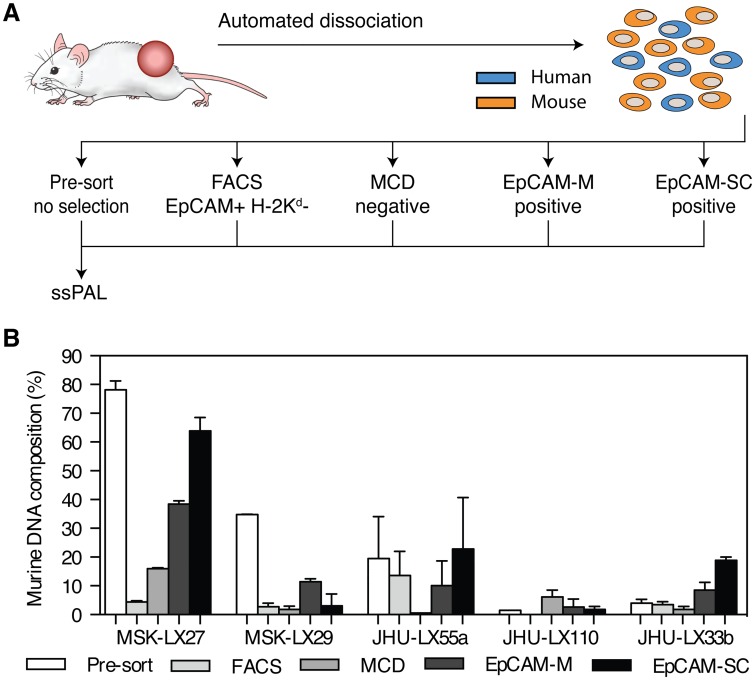
Comparison of cell separation techniques. (A) A schematic describing the experimental process used to test the four cell separation techniques. A single tumor from each PDX line was processed using a gentleMACS^™^ Octo Dissociator to obtain a single cell suspension comprising a heterogeneous mixture of human cells (blue) and mouse cells (orange). The suspension was divided in five samples. The pre-sort sample received no further processing. The remaining 4 tubes were processed using each method to obtain separate human and mouse cell populations. Each resulting human fraction and the pre-sort sample were analyzed using ssPAL. (B) ssPAL analysis results for primer pair 5 and 43 were averaged together to obtain the human DNA percentage for each separation method. After performing ssPAL analysis, results indicate that on average, MCD yields the highest sample purity over FACS, EpCAM-M and EpCAM-SC. The starting amount of murine stromal contamination also influences the effectiveness of the kit used, with EpCAM-M and EpCAM-SC performing poorly with higher initial murine content. Error bars represent standard error.

Since ssPAL analysis revealed a wide range of stromal contamination between our PDX lines, we sought to identify a commercial kit that could efficiently separate mouse and human components following tumor dissociation into a single cell suspension. We had previously established FACS as the gold standard for murine/human cell separation in our lab; however, this technique is expensive and time consuming. The Miltenyi mouse cell depletion kit (MCD), the STEMCELL technologies EasySep EpCam positive selection kit and the Miltenyi EpCam positive selection kit were tested for their ability to purify human cancer cells from dissociated PDX tumors compared to FACS. The characteristics of each cell separation are listed in Table 1. Tumors from multiple PDX lines were processed into a single cell suspension and enriched, followed by DNA extraction of the enriched human fraction and downstream ssPAL analysis Fig 4A. While the results showed that FACS yields the highest purity out of all methods in 2 out of 5 PDX lines, MCD clearly emerged as the most efficient technique to separate human and murine fractions irrespective of the initial level of murine cell contamination. The EpCAM selection kits from STEMCELL and Miltenyi yielded varying degrees of purity depending on the starting murine stromal fraction Fig 4B. Surprisingly, in some of the PDX lines EpCAM positive selection actually enriched the post-sort sample with murine cells instead of removing them Fig 4B. The STEMCELL and Miltenyi EpCAM positive selection kits were designed to purify cancer cells from human tissues and cell cultures, as well as to isolate circulating tumor cells from peripheral blood, stool samples and gastric aspirates. We speculate that it is possible that aggregates of mouse and human cells may form and that positive selection for human cells could cause the retention of the attached mouse cells, altering the final composition of the sample post purification. We observed that H-2K^d^/EpCAM double positive murine cells are present in our PDX tumor samples and could reflect species cross-reactivity of the primary antibody, which would be particularly problematic in the case of tumors with low EpCAM expression. These issues would not be as prevalent when using MCD since by targeting murine cells, as any mixed species aggregates or double positive cells would be removed, leaving behind only cells of human origin. These results highlight the variability of each approach and the importance of testing different methods of human cell enrichment to select the optimal technique for PDX tumor purification.

**Table 1:** Description of cell separation techniques.

Technique	Company	Marker for selection	Population targeted	Selection type
FACS	various	H-2K^d^ + EpCAM	mouse + human	sorting
MCD	Miltenyi	proprietary cocktail	mouse	negative
EpCAM-M	Miltenyi	EpCAM	human	positive
EpCAM-SC	STEMCELL	EpCAM	human	positive

### ssPAL analysis detects spontaneous murine malignancies

The generation of PDX requires the use of immunodeficient mice to avoid rejection of human tissue. A drawback of using immunodeficient animals is their increased prevalence of spontaneous tumorigenesis over wild-type mice. These mice develop a variety of malignancies over time, primarily lymphoma [12]. These cancers are easily transplantable over several passages, with a higher than 90% rate of engraftment and rapid growth rate. Due to this phenomenon in some cases what was thought to be a tumor derived from a human patient is actually a spontaneous mouse tumor. The species of origin of these malignancies can be hard to distinguish by morphology alone, and requires short tandem repeat (STR) analysis to confirm their species of origin, another expensive and time-consuming process. ssPAL analysis can be used as a first pass test to determine the species of origin of a tumor sample. Our results suggest that obtaining a murine contamination percentage higher than 95% by ssPAL analysis indicates a tumor is likely to be of mouse origin and should receive careful scrutiny. While analyzing PDX lines using ssPAL, we identified four PDX lines whose ssPAL score exceeded this cutoff line, and follow up testing using STR analysis subsequently confirmed they were not human tumors Table 2.

**Table 2:** ssPAL analysis detects spontaneous murine tumor formation.

	Primer Pair 5		Primer Pair 43		
PDX line	Murine DNA %	Human DNA %	Murine DNA %	Human DNA %	STR results
JHU-LX82	99.4	0.6	98.4	1.7	failed
JHU-LX21	96.6	3.4	100.0	0.0	failed
MSK-LX38	99.2	0.8	99.8	0.2	NA
NHJ29	94.5	5.5	97.0	3.0	failed

#### *In silico* modeling of mouse stromal admixture on next generation sequencing data

Genomic characterization has become increasingly critical to therapeutic analysis using PDX. Detailed information on somatic mutations and copy number changes is essential for selecting representative models and interpretation of experimental results. To assess the effects of stromal contamination on next generation sequencing data, we used an *in silico* modeling approach to generate virtual PDX sequencing data. High-depth Illumina Platinum Genome whole genome sequencing data and the Sanger Mouse Genome Project NSG mouse whole genome sequencing data were used to generate 3 types of virtual sample: virtual normal (NA12882 technical replicate 1; 30× DoC), virtual tumor (NA12882 technical replicate 2 with spiked in known mutations; 60× DoC), and virtual xenograft (60× DoC virtual tumor with reads randomly replaced by NSG mouse whole genome reads).

**Fig 5:**
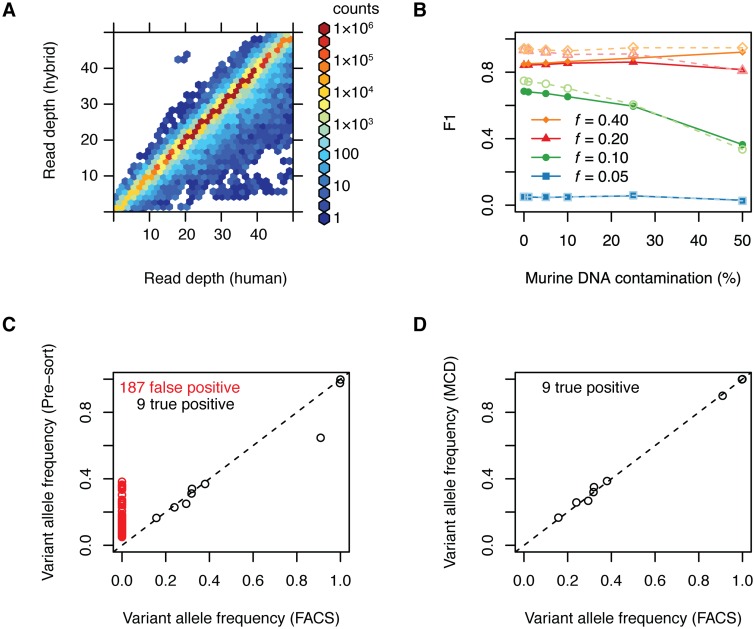
Effect of murine DNA contamination on next-generation sequencing. (A) Read depth of all bases of human chr22 after aligning reads from a 30× mean depth of coverage (DoC) WGSS experiment to either a hybrid human/mouse or human reference genome. (B) Mutation calling accuracy of a known set of mutations and variant frequencies (*f*) expressed as *F*-score (F1), the harmonic weighted average of precision and recall, as a function of of simulated mouse DNA contamination for WGSS (solid symbols) and WES (open symbols). (C) The MSK-LX29 PDX and patient matched normal DNA was sequenced using MSK-IMPACT and aligned to the human reference genome. Numerous false positive mutation calls were observed in the pre-sort sample when compared to FACS (red symbols). (D) After MCD, all false positive mutation calls are eliminated.

This data was then mapped to a hybrid human/mouse reference genome and reads mapping to mouse contigs were discarded. To estimate the potential consequences of loss of coverage due to multi-mapping reads, DoC was calculated for each base on human chr22 after aligning the virtual normal sample to either the human or hybrid reference genome. Coverage was reduced for only a minority of bases when aligning to the hybrid genome, consistent with a limited fraction of reads multi-mapping to human and mouse genomes; however, >98% of bases are covered at a depth of 15 or greater using either a hybrid or human reference genome Fig 5A. We therefore conclude that mapping to a hybrid genome does not cause significant gaps in coverage depth that would be expected to impair mutation calling sensitivity. After mapping the virtual tumor and virtual xenograft samples to the hybrid genome, MuTect was used to call mutations versus the matched virtual normal sample [32] Fig 5B. The primary effect of increasing mouse stromal admixture was loss of depth and consequently decreased power to detect subclonal mutations with low variant allele frequencies. This effect was present in both whole genome and exonic regions, although we note that mutation calling consistently performed better in exonic regions.

### High-depth targeted capture sequencing of patient derived xenografts

We performed high-depth targeted capture sequencing on a panel of 341 cancer related genes on patient-derived xenograft DNA from MSK-LX29 that had been purified by FACS or MCD using MSK-IMPACT [27]. The resulting raw reads were mapped to the human reference genome prior to mutation calling versus matched normal DNA from blood. Abundant false positive mutations were called in the pre-sort sample compared to samples purified by FACS at a rate 20-fold higher than true positive mutations (187 and 9, respectively) Fig 5C, while MCD purification was able to eliminate all false positive mutation calls Fig 5D. Although MSK-IMPACT uses capture probes designed for the human genome, we found that targeted capture can also pull down mouse DNA sequences, including some sequences that do not map to the human genome, increasing false positive mutation calls. When separately mapped to a hybrid reference genome, all samples called identical mutations to the FACS and MCD purified samples mapped to the human reference genome.

## Discussion

One of the primary barriers to successful drug development in oncology is the commonly observed discrepancy between therapeutic efficacy in preclinical models and in patients. There are many factors that may contribute to failure of preclinical models to predict clinical outcomes. Some investigators have stressed the lack of consistent standards and rigorous statistical methods in preclinical therapeutic assessment [35]. A larger issue may in fact be the models themselves—and the extent to which the models commonly used may not be adequately representative of the clinical disease. The PDX approach is one strategy for generating preclinical models very closely associated with the human disease.

Over the past decades it has become increasing clear that within any one histologically defined cancer type such as lung adenocarcinoma, there are in fact many distinct pathologic diseases defined by activation of distinct oncogenic drivers [36]. In developing representative preclinical models, it is therefore essential to develop robust methods for analyzing the genomic landscapes of those models. Unlike cell line-based xenografts, PDX cancer cells are in general never grown in the absence of mouse stromal components. The strategies we define here, to characterize the human:mouse cell ratio within PDX tumors, and to rapidly and reproducibly purify the human cell component for genomic profiling, will be of general utility across disease types in analyzing PDX lines. Notably, beyond the genomic analyses exemplified here, the same approaches could be applied to rapidly analyze gene expression or proteomic profiles of the malignant cell fraction of complex and multicellular PDX tumors.

Of the various methodologies tested for isolation of human cells from PDX tumors, ssPAL analysis suggests that two approaches, isolation by flow cytometric sorting (FACS) or by a magnetic bead-based mouse cell depletion (MCD) kit were consistently effective across a range of human:mouse cell ratios. Of these two, MCD may in many contexts be the preferred method, as it is rapid, consistently effective, simple, and does not require ready access to expensive equipment or instrument time. The other methods assessed, both dependent on positive selection for human EpCAM, appeared to be less effective in tumors with extensive mouse stromal content (e.g. MSK-LX27 in Fig 4B) where human tumor cell purification is most important. These observations are not necessarily surprising since the EpCAM positive selection kits were designed to purify cancer cells from human tissue samples and may not be the optimal method to separate mixed species fractions while the MCD kit was designed with the purpose of enriching human cells from xenograft tumor tissue. MCD has additional advantages over EpCAM positive selection as well. This technique can remove aggregates of human:mouse cells in a sample, which would be retained when using EpCAM selection, leading to incomplete purification. Additionally, a significant population of the H-2K^d^ positive murine cells can also express EpCAM and would presumably be pulled down by a positive selection kit. EpCAM has itself become a controversial marker for epithelial tumor cells. Previous studies have shown that EpCAM expression can be lost in epithelial tumor cells undergoing EMT and circulating tumor cells [37, 38]. Moreover, EpCAM is not uniformly expressed in all tumor types and may not provide the best target for human cancer cell selection [39]. By targeting the murine cells for removal, MCD offers a more reliable and consistent approach over EpCAM positive selection.

The observed stability in stromal content over multiple generations of PDX mice supports a model in which a homeostatic paracrine signaling mechanisms operant between cancer cells and stroma components sets a stromal content characteristic of each cancer line. A large number of tumor:stromal interaction pathways have been explored as possible therapeutic targets [40]. While many secreted and cell surface proteins have been shown to influence cancer-associated stromal biology, the factors establishing and maintaining the cancer:stromal cell ratio have not been well defined. Cancer associated “stroma” of course includes a complex mixture of cell types including connective tissue, hematologic, and vascular components, among others; this study did not seek to evaluate the stability of the composition of tumor-associated stroma over time. This could represent an interesting future direction.

Another open question is the extent to which the stable stromal content of a given PDX line is directly reflective of the stromal content of the human tumor of origin. We are unable to address this question specifically using the models presented here. The highest take rate for lung cancer xenografting in our experience has come from malignant pleural effusions (lacking a fixed stromal structure), and in the cases of biopsy-derived xenografts, the amount of available human tumor material for analysis is limited. Addressing this question will require a larger dataset focused on a series of models for which paired primary tumor biopsy material or surgically resected human tumor is available. Identifying commonalities and differences in tumor cell interaction with human versus mouse stromal components could further support, or define important limitations, of PDX as an approach to modeling human tumor biology.

Our data on species-specific separation and genetic analysis also highlights the importance of vigilance in monitoring PDX lines for possible overgrowth and replacement by spontaneous mouse tumors, such as murine lymphoma. We identified four such events in PDX derived in our own laboratory and others. This observation emphasizes the need for serial reassessment and confirmation of the identity of human tumors maintained in highly immunosuppressed mice. A tumor analyzed by ssPAL where the mouse cell content exceeds 95% should raise an immediate red flag.

The techniques described here outline dependable approaches to defining the mouse to human cell content of PDX tumor models, and to separating these components for species-specific analyses including high fidelity mutation profiling. Both the *in silico* modeling and the actual PDX sequencing performed here highlight the importance of this separation in minimizing false negative and false positive mutation calls in genomic characterization of these models. We believe the techniques of ssPAL for assessment of stromal content and MCD for isolation of human tumor cells for cancer cell-specific molecular profiling will be of general utility to the increasing community of investigators using PDX models of human tumor biology.
